# Life satisfaction of women of working age shortly after breast cancer surgery

**DOI:** 10.1007/s11136-016-1479-z

**Published:** 2017-01-09

**Authors:** Mariann Olsson, Marie Nilsson, Kerstin Fugl-Meyer, Lena-Marie Petersson, Agneta Wennman-Larsen, Linnea Kjeldgård, Kristina Alexanderson

**Affiliations:** 1 Division of Social Work, Department of Neurobiology, Care Sciences and Society, Karolinska Institutet, Stockholm, Sweden; 2 Department of Social Work, Karolinska University Hospital, Stockholm, Sweden; 3 Division of Insurance Medicine, Department of Clinical Neuroscience, Karolinska Institutet, Stockholm, Sweden; 4 Sophiahemmet University, Stockholm, Sweden

**Keywords:** Breast cancer, Quality of life, Life satisfaction, Social support

## Abstract

**Purpose:**

To explore, among women of working age, satisfaction with life as a whole and with different life domains, and its associations with social and health variables, shortly after breast cancer surgery.

**Methods:**

This cross-sectional study included 605 women, aged 20–63 years, who had had breast cancer surgery with no distant metastasis, pre-surgical chemotherapy, or previous breast cancer. Associations between LiSat-11 and demographic and social factors as well as health- and treatment-related variables were analysed by multivariable logistic regression.

**Results:**

Compared with Swedish reference levels, the women were, after breast cancer surgery, less satisfied with life, particularly sexual life. Women working shortly after breast cancer surgery were more often satisfied with life in provision domains compared with the reference population. Although most included variables showed associations with satisfaction, after adjustment for all significantly associated variables, only six variables—having children, being in work, having emotional and informational social support, and having good physical and emotional functioning—were positively associated with satisfaction with life as a whole. The odds ratios for satisfaction were higher in most life domains if the woman had social support and good emotional and cognitive functioning.

**Conclusions:**

One month after breast cancer surgery, satisfaction with different life domains was associated primarily with social support and health-related functioning. However, this soon after surgery, treatment-related variables showed no significant associations with life satisfaction. These results are useful for planning interventions to enhance e.g. social support and emotional as well as cognitive functioning.

## Introduction

Life satisfaction is a concept that characterises affect—an emotion [1, 2]—and, as stated by von Wright [3], when the level of satisfaction is brought to consciousness, the individual relates hedonic affect to internalised roles. Satisfaction may be domain specific or characterise life as a whole. Thus, the self-reported level of life satisfaction characterises the contentment which an individual derives from a certain domain of life or from life as a whole, and can be interpreted as a social indicator [4].

For breast cancer, the concept of health-related quality of life is often used when aiming to determine and understand individuals’ well-being [5]. This concept concerns how physical, mental and social functions are affected by the disease, with most women with breast cancer being found to return to the same level of quality of life as the general population after the end of treatment [6]. Life satisfaction is most often considered as a separate aspect of quality of life, reflecting an individual’s appraisal of life in an aspiration–goal achievement model rather than functional limitations [2, 7].

An individual’s own aspirations as well as external demands on them are dynamic and may change and develop over time and with context. An individual’s level of overall life satisfaction varies over time, but it is quite constant in larger populations [4]. Satisfaction with different domains of life has been used as a key outcome regarding recovery from disease and an indicator for adaption to new life conditions [8–10]. Furthermore, life satisfaction has recently been used to investigate associations with breast cancer [11]. In the cited Chinese screening programme, life satisfaction was associated with healthy lifestyle and with lower detection rate of breast diseases.

In studies of general populations, life satisfaction has mainly been found to be gender independent [2], but associated with age, educational level, work status, perceived health [12, 13] and social support [14, 15]. Qualitative studies show how their own preferences and goals are important for women after breast cancer surgery to guide their own actions as well as to form assessments of encounters with different stakeholders during the cancer trajectory [16, 17].

Exploring life satisfaction in breast cancer survivors is thus necessary to understand in which areas of life women experience imbalance between personal goals and current life conditions and in which areas they might need compensatory or complementary information and/or support. However, although there are several quality-of-life studies of breast cancer patients internationally [5, 18, 19], life satisfaction shortly after breast cancer surgery has, to the best of our knowledge, hitherto only been the focus of one small study of breast cancer patients [20], in which chemotherapy showed strong negative associations with life satisfaction. Furthermore, no study has been performed concerning different life domains and their associations with socio-demographic factors, work conditions, social support or health.

The present study aimed to explore satisfaction with life as a whole and with different life domains, and its association with social and health variables, at 1–2 months after breast cancer surgery.

## Methods

A cohort of women of working age who had had breast cancer surgery at three hospitals in Stockholm, Sweden during the period of June 2007 to November 2009 was formed as part of a larger study. The women were included consecutively, at the follow-up visit at the oncology clinic, which usually takes place about 3 weeks after surgery. Inclusion criteria were: age 20–63 years, living in Stockholm County, literate in Swedish. Exclusion criteria were: known distant metastasis, pre-surgical chemotherapy, and/or previous breast cancer diagnosis. A comprehensive questionnaire was developed and repeatedly distributed on six occasions. After oral and written information about the study, the women received the questionnaire and a prepaid envelope. In all, 970 women met the inclusion criteria, but 48 (4.9%) were not invited due to administrative failures. Of the invited women, 725 (78.5%) agreed to participate, gave their informed consent, and returned the questionnaire. Further information about the cohort has been published previously [21].

In the present cross-sectional study, baseline questionnaires from women who were in paid work (full- or part-time) at time of diagnosis (*n* = 605) were included. Social variables that, according to the above-reviewed literature, are associated with life satisfaction were analysed: (1) socio-demographics (age, marital status, having children, country of birth), (2) work-related conditions, (3) social support and (4) health- and treatment-related variables (type of surgery and planned chemotherapy).

### Analysed variables

*Life satisfaction* was measured using the Life Satisfaction Checklist-11 (LiSat-11), a generic and validated tool comprising 11 items [2] which has been used in connection with different diseases e.g. stroke [14, 22], and traumatic injuries [23, 24] including multiple trauma [25]. It has also been used in connection with different cancers [20, 26, 27]. LiSat-11 includes one item regarding satisfaction with life as a whole, and ten items regarding satisfaction with different domains of life, forming four different factors [2]: (1) provision (satisfaction with vocational situation and economy), (2) spare time (satisfaction with leisure and contacts with friends and acquaintances), (3) closeness (satisfaction with sexual life, partner relationship and family life) and (4) health (satisfaction with physical health, psychological health, and P-ADL = personal activities of daily living). Each item is scored on a six-point scale: 1 = very unsatisfied, 2 = unsatisfied, 3 = rather unsatisfied, 4 = rather satisfied, 5 = satisfied, 6 = very satisfied. The answers were dichotomised into “satisfied” (“very satisfied” or “satisfied”) and “not satisfied” (from “rather satisfied” to “very unsatisfied”), in line with recommendations [2]. Norm data from a Swedish nationally representative population presented in two different studies were used for general comparison. The first study [2] covered life satisfaction of 2533 individuals (1326 men and 1207 women) aged 18–64 years. The second [28] concerned life satisfaction of 926 of the women, i.e. those reporting a steady partner relationship.

#### Socio-demographics

In the statistical calculations, age was dichotomised by the median, country of birth into “Sweden” and “outside Sweden”, marital status into “married” and “not married”, having children into “yes” or “no” (regardless of the age of these children), educational level into “low” (elementary school or grammar/secondary school <12 years) and “high” (college/university ≥12 years) and experiencing financial hardship into “yes” and “no”.

#### Work conditions

Work status was measured with a question regarding sickness absence or not at baseline; women not on full-time sickness absence were classified as in paid work. Two more variables were chosen, since they were shown in our previous work to be of importance for returning to work after breast cancer surgery and thus of potential importance for satisfaction at least with vocational life [29]: strenuous work posture and perceived work adjustment.

Strenuous work posture was measured using three questions “Do you have to work with your arms above your shoulders or below your knees?”, “Do you have to work in a bent or twisted position, or in any other inappropriate posture?”, and “Does your job require heavy lifting?”. The response options ranged from “rarely/never” (=1) to “very often/always” (=5). A “work posture” index was created as described by Nilsson et al. [30] by taking a summed average, where a minimum of two items had to be answered; this index was dichotomised based on the response options into “no” (<3.0) and “yes” (≥3.0).

Perceived work adjustment was measured using six items. The following three stem from the Adjustment Latitude scale [31]: “When the work you do becomes physically too strenuous, is it possible for you to slow the pace or perform your duties in some other way?”; “When the work you do becomes too psychologically strenuous, is it possible for you to influence your situation?”; “In what way can you adjust your work situation if you are not feeling well. Can you decide yourself which tasks to perform?”. The response options were “always” (=3), “sometimes” (=2), “seldom/never” (=1) and “not applicable” (=0). Furthermore, the following three items from the National Working Life Cohort [32] were included: “Can you set your own work pace?”; “Can you to some extent decide when various tasks are to be done?”; “Are you partly/sometimes allowed to participate in the planning/organisation of your work?”. The response options were “always” (=3), “usually” (=2), “seldom” (=1) and “never” (=0). “Seldom” and “never” were collapsed into “seldom/never” to correspond to the Adjustment Latitude scale. An index was calculated [30], then dichotomised by the median into high (>2) versus low (≤2).

#### Social support

We wanted to obtain information about perceived social support in the primary network as well as at work. Perceived social support in the primary network was measured using two questions from the Social Support Short Form (SS-13) instrument [33]: one to indicate emotional support and the other to measure instrumental support, e.g. someone who is ready to give advice when needed. The answers were dichotomised to “no” (“no”, “not sure”, and “yes, maybe”) and “yes” (“yes” and “I’m sure”).

Perceived social support at work was measured using two single items from the National Working Life Cohort [32]: “Are you able to get support and encouragement from colleagues when you feel that things aren’t going well at work?” and “Are you able to get support and encouragement from your immediate supervisor when you feel that things aren’t going well at work?”. The response options were: “always” (=3), “usually” (=2), “seldom” (=1) and “never” (=0). The items were dichotomised by the median as “highly supportive” (3+) versus “less supportive” (≤2).

#### Health

Physical, emotional and cognitive functioning were investigated using the corresponding scales from the European Organisation for Research and Treatment of Cancer (EORTC) quality-of-life core questionnaire QLQ C30 [34], a measure with Swedish reference values [35]. The physical functioning (PF) scale consists of five items regarding problems doing strenuous activities, taking a long or short walk, having to stay in bed or a chair during the daytime or needing help with daily activities. The emotional functioning (EF) scale consists of four items regarding feeling tense, worried, irritable or depressed. The cognitive functioning (CF) scale consists of two items concerning memory or concentration problems. All items have the following response alternatives: “not at all”, “a little”, “quite a bit” and “much”. The responses were summed and divided by the number of items in each scale, creating average summated scales based on a minimum of 50% of the items responded to in each scale. The raw scores for each scale were then transformed into a 0–100 scale [36], with 0 considered as poor functioning and 100 as excellent functioning. Cronbach’s *α* was 0.69 for physical functioning, 0.85 for emotional functioning and 0.75 for cognitive functioning. For the analyses, the scales were dichotomised into “less than good” or “good” by the median for each scale (PF median = 87, EF median = 67, CF median = 84).

Data on type of breast surgery (breast-conserving surgery or mastectomy) and planned postoperative chemotherapy (yes or no) were obtained from the Swedish National Quality Register for Breast Cancer,^1^ a register with high validity [37].

### Statistical analyses

Characteristics of the women are reported as frequencies and percentages. Differences in proportions of high LiSat scores between groups (formed by dichotomisation of variables under study) were analysed using Pearson *χ* ^2^ tests. The level of statistical significance was specified to be <0.05. Variables significantly associated with satisfaction in each of the life domains were kept in multivariable analyses of that specific domain. These multivariable logistic regression analyses (stepwise backward procedure) were employed to calculate odds ratios (OR) with 95% confidence intervals (CI) for satisfaction in each life domain, controlling for only covariates with statistically significant results in the *χ* ^2^ tests. In each step, backward elimination of the one variable with the highest *p* value was performed until all remaining variables were significantly associated with the item. Statistical analyses were performed using IBM SPSS Statistics 20.

## Results

Socio-demographics, work conditions, social support and health/treatment-related characteristics of the participants are presented in Table 1. The median age of the women was 52 years (range 26–63 years); slightly more than half of them were married and had a college or university education (Table 1). More than half of the women were in paid work when completing the questionnaire, and more than one-third had a job that they were able to adjust according to the demands of their health condition. A minority perceived high levels of social support at work, from colleagues more often than from supervisors. However, more than 80% had someone close for emotional and instrumental support. Emotional and cognitive functioning were less good (*m* = 67 versus 83 and *m* = 83 versus 89) compared with Swedish female norm material [35]. Mastectomy was performed in one-third of surgeries, and postoperative chemotherapy was planned for half of the women (Table 1).

**Table 1:** Socio-demographic, work-related, social support and health/treatment-related characteristics of the women (*n* = 605)

Socio-demographic variables^a^	
Age (years)	
Mean (SD)	51.1 (7.9)
Median (range)	52 (6–63)

	*n*	(%)^b^
Age		
Below 52 years old	295	48.8
52 years or older	310	51.2
Country of birth		
Sweden	517	85.5
Other	85	14.0
Married		
Yes	329	54.4
No	267	44.1
Have children		
Yes	519	85.8
No	84	13.9
Educational level		
Low (<12 years)	254	42.0
High (≥12 years)	350	57.7
Financial hardship		
No	448	74.0
Yes	152	25.2
Work-related variables^a^		
In work		
Yes	311	51.5
No	281	46.4
Strenuous work posture		
No	475	78.5
Yes	119	19.7
Work adjustment		
Low	368	60.8
High	227	37.5
Social support variables^a^		
Social support from colleagues		
Low	395	65.3
High	197	32.6
Social support from supervisors		
Low	418	69.0
High	157	26.0
Emotional support		
No	94	15.5
Yes	511	84.5
Instrumental support		
No	111	18.3
Yes	494	81.7
Health-related variables^a^		
Physical function		
Less than good	315	52.1
Good (≥87)	290	47.9
Emotional function		
Less than good	322	53.2
Good (≥67)	281	46.5
Cognitive function		
Less than good	370	61.2
Good (≥84)	233	38.5
Treatment-related variables^a^		
Type of breast surgery		
Breast-conserving surgery	408	67.4
Mastectomy	197	32.6
Planned chemotherapy		
Yes	287	47.4
No	317	52.4

^a^Variables dichotomised as described in text

^b^Missing values not shown, so percentages may sum to <100

### Life satisfaction

**Fig. 1:**
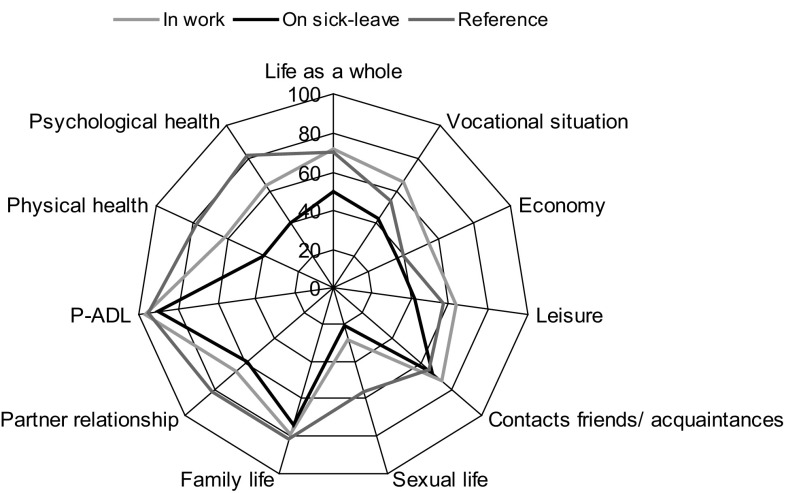
Satisfaction with life in different domains of life (% satisfied) in three groups: in work, on sick leave shortly after breast cancer surgery, and norm data (women in Sweden aged 18–65 years and reporting a partner relationship) [28]

Shortly after breast cancer surgery, women reported significantly less satisfaction with physical and psychological health as well as with partner relationship and, in particular, sexual life compared with a Swedish reference population [2, 28]. Satisfaction with life was found more often among those in paid work than among those on full-time sick leave regarding life as a whole and in the life domains of provision and health (with the exception of P-ADL; Fig. 1). Working women with breast cancer were even more often satisfied with life in the provision domains compared with the reference population.

Variables that, according to the bivariate analyses, pointed at significant differences in satisfaction with life as a whole or with any life domain were all socio-demographic—most work-related variables, all social support and all health- (and treatment) related variables—but they showed a highly variable pattern for the different life domains (see Appendix).

### Life satisfaction as dependent variable in multivariable regressions

Results from the multivariable logistic regression models of satisfaction with life as a whole and in each domain are presented in Table 2. The OR for satisfaction with life as a whole, adjusted for all included variables, was higher when having children, being in work, having available social support and having good physical and emotional functioning. Cognitive functioning did not predict high OR for satisfaction with life as a whole, nor were the treatment variables—type of surgery and planned chemotherapy—significantly associated with higher OR for satisfaction with life as a whole in the final multivariable model.

**Table 2:** Multivariable associations between socio-demographic, work-related, social support and health-related variables and the dependent variables, i.e. satisfaction with life as a whole and with life in different domains

	Life as a whole	Provision		Spare time		Closeness			Health		
		Vocational situation	Economy	Leisure	Contacts with friends	Sexual life	Family life	Partner relationship	P-ADL	Physical health	Psychological health
Socio-demographics											
≥52 years old				1.5 (1.0–2.3)							
Born outside Sweden									0.4 (0.2–0.9)		
Married			1.5 (1.0–2.3)				2.0 (1.3–3.2)	4.6 (3.1–7.0)			
Have children	2.5 (1.4–4.3)					2.6 (1.2–5.4)	2.6 (1.4–4.6)				2.0 (1.1–3.7)
Education ≥12 years			1.6 (1.0–2.4)								
Financial hardship		0.5 (0.3–0.8)	0.1 (0–0.1)			0.5 (0.3–0.9)		0.5 (0.3–0.8)			
Work conditions											
In work	1.9 (1.3–3.0)	1.7 (1.1–2.5)	1.6 (1.0–2.4)								
Strenuous work			0.4 (0.2–0.7)	0.6 (0.3–0.9)							
High work adjustment		2.0 (1.3–3.1)									1.7 (1.1–2.7)
Social support											
Emotional support	3.0 (1.5–6.0)						3.2 (1.6–6.3)	4.1 (2.0–8.4)	2.3 (1.1–5.1)		2.9 (1.6–5.4)
Instrumental support	3.2 (1.7–6.1)	2.1 (1.3–3.5)	3.0 (1.7–5.4)	7.8 (4.2–14.4)	5.6 (3.5–9.1)	4.5 (2.0–10.0)	3.1 (1.6–5.9)	2.3 (1.2–4.5)		2.4 (1.4–4.2)	
Support from colleagues				2.0 (1.3–3.0)	1.6 (1.1–2.6)	1.7 (1.1–2.5)					1.8 (1.1–2.8)
Support from supervisors		2.0 (1.3–3.2)	1.8 (1.1–2.8)							2.0 (1.3–3.1)	
Health											
Good physical functioning	1.9 (1.2–2.8)	1.6 (1.1–2.5)		2.4 (1.6–3.7)					5.8 (1.9–7.4)	3.0 (2.0–4.5)	
Good emotional functioning	3.6 (2.4–5.5)			1.8 (1.2–2.8)	2.0 (1.3–3.2)		1.9 (1.2–3.2)			2.0 (1.3–3.0)	7.1 (4.6–10.9)
Good cognitive functioning		2.2 (1.5–3.4)	1.9 (1.2–2.9)	1.8 (1.2–2.8)	2.0 (1.3–3.2)		2.0 (1.2–3.3)	1.9 (1.2–2.9)		1.7 (1.1–2.6)	2.7 (1.8–4.2)

Odds ratios with 95% confidence intervals. Only significant associations (*p* < 0.05) are shown

Regarding satisfaction with life in the different life domains, the OR patterns varied; no single variable was, when adjusting for all included variables, associated with higher OR for satisfaction with life in all life domains. Socio-demographics were associated with higher OR for life satisfaction in a few domains, whereas working was, when adjusted for all included variables, associated only with satisfaction with life in the provision domains and with satisfaction with life as a whole.

However, having social support was positively associated with OR for satisfaction in most life domains, and having instrumental social support was the variable showing the highest OR for life satisfaction (OR = 7.8 for satisfaction with leisure time). A minority of women perceived high levels of social support at work. Those with support from supervisors had higher OR for satisfaction with life in provision domains, while support from colleagues was associated with satisfaction with life in the spare time domains. Good health, especially emotional and cognitive functioning, was associated with satisfaction with life in most domains.

## Discussion

This is one of the few studies about life satisfaction shortly after onset of a serious disease and about life satisfaction among cancer patients. Compared with a Swedish reference group [2, 28], women shortly after breast cancer surgery experienced less satisfaction with life as a whole, with sexual life and partner relationship, as well as with health. A higher rate of the women working shortly after breast cancer surgery showed satisfaction with life in the vocational and financial domains than those not working; the rate was even higher than in the reference group. Moreover, satisfaction with life as a whole was associated with work status, social support and health functioning—but not with socio-demographics or other work- or treatment-related variables.

### Social variables and life satisfaction

In the present study, women in work were more satisfied with life than those who were absent due to sickness. However, being in work shortly after breast cancer showed few significant associations with satisfaction in separate life domains when adjusting for other included variables. Thus, only satisfaction with life as a whole and with life in provision domains was significantly associated with working or not. It seems that, in our study, work and work capacity, facilitated by non-strenuous work posture and adjustable work conditions, could, soon after breast cancer surgery, function as a most appreciated lifeline when other life domains are more taxing.

Not surprisingly and as expected, associations between financial hardship and dissatisfaction in the economy domain were confirmed. However, other expected associations were not found among these women, e.g. regarding education level and satisfaction with health or with life as a whole, where one might expect that women with higher education would be more satisfied with life as a whole and with health [12, 13]. However, this study concerned a quite well-educated group of women, which may have biased the results.

The strong positive association between social support and life satisfaction was expected from knowledge about breast cancer and social support [38, 39]. This is also confirmed in research on life satisfaction among other patient groups and family caregiver groups [14, 40]. In our study, a minority of the women perceived high levels of support from supervisors and colleagues, in spite of the importance of these support sources for working [30] and life satisfaction. Moreover, perceived social support at work and vocational satisfaction have been shown to be associated not only shortly after breast cancer surgery [30] but also during the following two years [41].

### Health variables and life satisfaction

Given our theoretical position that life satisfaction is a concept of contentment stemming from the life domain in focus while quality of life concerns roles and functioning in that life domain, it is of special interest to discuss associations between the two. Not surprisingly, higher odds of life satisfaction with physical and psychological health were found in women with better HRQoL measured by physical and emotional functioning (QLQ-C30). Good emotional functioning was thus associated with satisfaction with life as a whole; however, this health-related quality-of-life aspect was not associated with satisfaction in the provision domains of life. An important finding was the association between cognitive functioning and satisfaction with life in most of the domains. Cognitive problems are frequently reported as a consequence of breast cancer treatment [42, 43], but these aspects have hitherto not been studied shortly after surgery. This is a source of dissatisfaction which the woman and her workplace might need specific information about, as also previously pointed out in a focus group study [16].

Similar to previous studies on life satisfaction among female patients [28], this study showed less satisfaction with sexual life. However, satisfaction with this life domain was not associated with health variables. It is suggested by the early work of Gyllensköld [44] and later research [45–47] that women’s self-image may be threatened by a mastectomy. Karabulut and Erci [48] found that women who received chemotherapy after mastectomy were significantly less satisfied with their sexual life. In the present study, no treatment-related variables were associated with life satisfaction when adjusting for all included variables. This difference in relation to previous research [20, 48] may be due to the timing of the investigation. Although planned chemotherapy was most often initiated at the time of our data collection, this early in the cancer trajectory, most consequences of chemotherapy treatment have not yet been experienced. Another possibility is that the cancer diagnosis per se—in this study an experience shared by all women—is associated with less life satisfaction and thus obscures associations with treatment-related variables.

### Limitations of the study

A limitation of the current study is the lack of a matched comparison group to this population of rather homogeneous and well-educated women. This is particularly important given the paucity of other studies on life satisfaction in breast cancer populations. Since the data relate to women of working age and with earlier stages of cancer, the results may not be representative of all women with breast cancer early after breast cancer surgery. However, Swedish norm data were available for comparisons regarding level of life satisfaction, and the response rate was high [21].

### Conclusions

One month after breast cancer surgery, satisfaction with life as a whole was associated with work status, social support and health-related functioning, especially cognitive functioning. Instrumental social support and cognitive functioning were positively associated with satisfaction with life in most separate life domains and should, therefore, be a focus of assessments. However, this soon after surgery, work conditions showed limited associations and treatment-related variables no associations with life satisfaction.

### Implications

The concept of life satisfaction helps us to understand in which areas of life women with breast cancer experience an imbalance between personal goals and current life conditions and where they might need support from health or social care workers or employers. We hope that the results of this study will be used to increase awareness about such individually experienced imbalances and to offer suggestions regarding possible treatments or educational and supportive interventions aiming to strengthen social support both in the primary network and in the workplace environment. Women’s emotional functioning should also be seen as crucial for life satisfaction and, when needed, merit proper treatment and/or support. Another area where it is implied that the woman and her workplace might need specific information in order to enhance life satisfaction and adjustment in several areas of the woman’s life situation is her cognitive functioning.

The failure to detect associations with treatment-related variables in the present study calls for continued analyses of life satisfaction over time in breast cancer patients, as suggested by Spagnola et al. [47], as well as in the case of general quality of life research [19], similar to the case of life satisfaction among patients with other chronic diseases. For example, in a progressive disease such as rheumatoid arthritis (RA), no significant associations [49] have been found between disease activity and satisfaction with life as a whole. Moons et al. [7], however, argue that life satisfaction should be considered a dynamic concept since both expectations/goals in life and experiences may change over time.

## Appendix

See Table 3.

**Table 3:** Percentage of women who were satisfied with life as a whole and in different domains

	Life as a whole (%)	Provision		Spare time		Closeness			Health					
		Vocational situation (%)	Economy (%)	Leisure (%)	Contacts with friends (%)	Sexual life (%)	Family life (%)	Partner relationship (%)	P-ADL (%)		Physical health (%)		Psychological health (%)	
Socio-demographics														
Age														
<52 years	59.7	**49.8**	**40.1**	**48.8**	69.0	24.8	75.4	61.1	93.2		49.7		**46.8**	
≥52 years	61.3	**58.6**	**50.2**	**57.5**	70.6	22.9	76.1	63.8	93.5		51.5		**56.6**	
Country of birth														
Sweden	***63.3***	55.8	46.9	***55.9***	***72.0***	25.0	77.0	62.9	***94.8***		52.1		**53.6**	
Other	***43.5***	44.7	35.7	***37.3***	***57.1***	16.5	68.3	60.5	***84.5***		41.7		**40.5**	
Married														
No	***54.4***	**49.8**	***35.8***	48.9	67.0	**19.6**	***66.4***	***42.5***	93.2		48.3		49.8	
Yes	***64.9***	**58.2**	***53.0***	56.7	71.6	**27.5**	***83.2***	***78.0***	93.6		52.3		54.0	
Having children														
No	***46.3***	48.8	41.7	47.6	66.7	**12.2**	***59.3***	***46.3***	92.9		45.2		**39.3**	
Yes	***62.8***	55.2	45.8	54.2	70.3	**25.4**	***78.4***	***65.0***	93.4		51.6		**53.9**	
Education														
Low (<12 years)	62.8	**49.2**	***36.8***	52.2	70.9	24.0	77.3	65.7	92.9		52.0		56.3	
High (≥12 years)	58.7	**57.9**	***51.3***	53.9	69.1	23.5	74.6	60.0	93.7		49.7		48.4	
Financial hardship														
No	***65.2***	***60.0***	***58.1***	***58.4***	***73.2***	***27.4***	78.1	***68.7***	94.4		***53.9***		***56.3***	
Yes	***48.0***	***38.2***	***7.3***	***38.9***	***61.6***	***14.6***	70.2	***43.9***	90.1		***41.1***		***39.7***	
Work-related variables														
In paid work														
Yes	***71.7***	***65.0***	***53.6***	***63.3***	73.0	27.5	78.6	66.2	**96.8**		***61.0***		***63.2***	
No	***49.3***	***42.5***	***35.7***	***42.4***	66.8	20.6	73.2	57.5	**90.4**		***39.3***		***40.1***	
Strenuous work posture														
No	**63.2**	***58.4***	***50.1***	***57.0***	70.5	22.6	75.6	61.1	***94.9***		**52.3**		**54.1**	
Yes	**52.1**	***41.2***	***28.0***	***39.8***	68.9	28.0	75.9	67.3	***87.3***		**39.8**		**43.2**	
Work adjustment														
Low (≤2)	***55.1***	***45.2***	***39.3***	***47.0***	**66.5**	23.5	74.5	**58.6**	92.9		***42.8***		***43.8***	
High (>2)	***69.6***	***70.9***	***55.6***	***63.0***	**75.8**	24.5	77.1	**68.3**	94.3		***61.9***		***64.8***	
Social support														
Emotional support														
No	***24.7***	***33.0***	***30.1***	***22.8***	***34.4***	***6.6***	***35.5***	***21.5***	***85.1***		***29.8***		***23.4***	
Yes	***67.1***	***58.2***	***48.0***	***58.8***	***76.3***	***27.0***	***83.2***	***70.1***	***94.9***		***54.4***		***57.1***	
Instrumental support														
No	***25.5***	***31.5***	***23.4***	***14.4***	***31.8***	***6.5***	***42.2***	***31.2***	90.0		***25.5***		***26.4***	
Yes	***68.4***	***59.4***	***50.2***	***62.1***	***78.3***	***27.8***	***83.2***	***69.5***	94.1		***56.2***		***57.5***	
Support from colleagues														
Low	***56.6***	***48.7***	**42.1**	***46.7***	***65.2***	***19.7***	73.3	**59.4**	93.4		**46.7**		***47.3***	
High	***69.4***	***67.0***	**52.6**	***66.3***	***79.7***	***32.1***	80.5	**69.3**	93.4		**57.1**		***61.2***	
Support from supervisors														
Low	***55.1***	***48.2***	***40.4***	***47.7***	***66.2***	***20.0***	***71.6***	***56.9***	93.0		***43.8***		***46.9***	
High	***73.1***	***72.0***	***59.0***	***67.3***	***79.6***	***33.6***	***83.3***	***73.9***	93.6		***64.3***		***63.5***	
Health-related variables														
Physical functioning														
Less than good	***47.6***	***42.7***	***35.8***	***37.4***	***63.1***	***18.4***	***70.3***	***56.3***	***88.5***		***32.3***		***40.3***	
Good (≥87)	***74.4***	***66.9***	***55.6***	***70.2***	***77.2***	***29.7***	***81.6***	***69.1***	***98.6***		***70.3***		***64.4***	
Emotional functioning														
Less than good	***43.0***	***43.0***	***38.6***	***40.4***	***60.1***	***18.2***	***66.4***	***53.2***	***91.0***		***36.6***		***28.1***	
Good (≥67)	***80.9***	***66.9***	***52.5***	***67.9***	***81.1***	***30.2***	***86.7***	***73.0***	***96.4***		***66.5***		***78.9***	
Cognitive functioning														
Less than good	***51.2***	***43.4***	***37.0***	***42.9***	***62.1***	***20.0***	***68.7***	***54.8***	***91.4***		***40.5***		***37.9***	
Good (≥84)	***75.3***	***71.2***	***58.0***	***69.7***	***82.4***	***29.9***	***87.3***	***74.9***	***97.0***		***66.7***		***74.0***	
Treatment-related variables														
Type of breast surgery														
Breast conserving	62.8	**57.1**	47.5	**56.7**	69.3	22.9	74.4	61.1	94.6		53.2		53.6	
Mastectomy	55.8	**48.5**	40.6	**46.1**	71.1	25.6	78.5	65.3	90.9		45.2		48.2	
Planned chemotherapy														
No	**65.2**	57.7	46.8	***58.4***	68.1	27.1	74.2	61.4	94.9		52.1		***58.2***	
Yes	**55.2**	50.3	43.4	***47.3***	71.7	20.3	77.4	63.5	91.6		49.1		***44.9***	
Norm data—Swedes of working age [2]	70	54	39	57	65	56	81	82	95		77		81	
Norm data—Swedish females [28]	75	54	40	58	68	66	86	83	98		78		82	

Bivariate analyses of distribution over socio-demographic, work-related, social support, health- and treatment-related characteristics

Total numbers in each column may vary (from *n* = 599 to *n* = 605). Pearson chi-square tests. Significance of differences indicated by use of fonts: *NS* regular font; *p* < 0.05 bold; *p* < 0.01 bold italics

## Footnotes

1 These data are used with the permission of the Steering Board of the Swedish National Quality Register for Breast Cancer.
